# Development of High-Cell-Density Tissue Method for Compressed Modular Bioactuator

**DOI:** 10.3390/mi13101725

**Published:** 2022-10-12

**Authors:** Takuto Nomura, Masaru Takeuchi, Eunhye Kim, Qiang Huang, Yasuhisa Hasegawa, Toshio Fukuda

**Affiliations:** 1 Department of Micro-Nano Mechanical Science and Engineering, Nagoya University, Nagoya 4648603, Japan; 2 Intelligent Robotics Institute, School of Mechatronical Engineering, Beijing Institute of Technology, Beijing 100081, China

**Keywords:** biorobot, bioactuator, cultured muscle

## Abstract

Bioactuators have been developed in many studies in the recent decade for actuators of micro-biorobots. However, bioactuators have not shown the same power as animal muscles. Centrifugal force was used in this study to increase the cell density of cultured muscle cells that make up the bioactuator. The effect of the centrifugal force on cells in the matrix gel before curing was investigated, and the optimal centrifugal force was identified to be around 450× *g*. The compressed modular bioactuator (C-MBA) fabricated in this study exhibited 1.71 times higher cell density than the conventional method. In addition, the contractile force per unit cross-sectional area was 1.88 times higher. The proposed method will contribute to new bioactuators with the same power as living muscles in animals.

## 1. Introduction

Biorobots are new robots that are expected to combine living organisms’ characteristics with machines’ controllability and precision [1]. Bioactuators are actuators that are based on a living muscle tissue. A bioactuator is expected to provide the robot with the characteristics of biological muscles, such as energy efficiency and elasticity. These bioactuators are intended to be applied in an environment similar to the human body because of their elasticity, redundancy, and biocompatibility. These environments are, for example, prosthetic limbs, microrobots that move inside the body, and alternative models for pharmacology.

**Figure 1:**
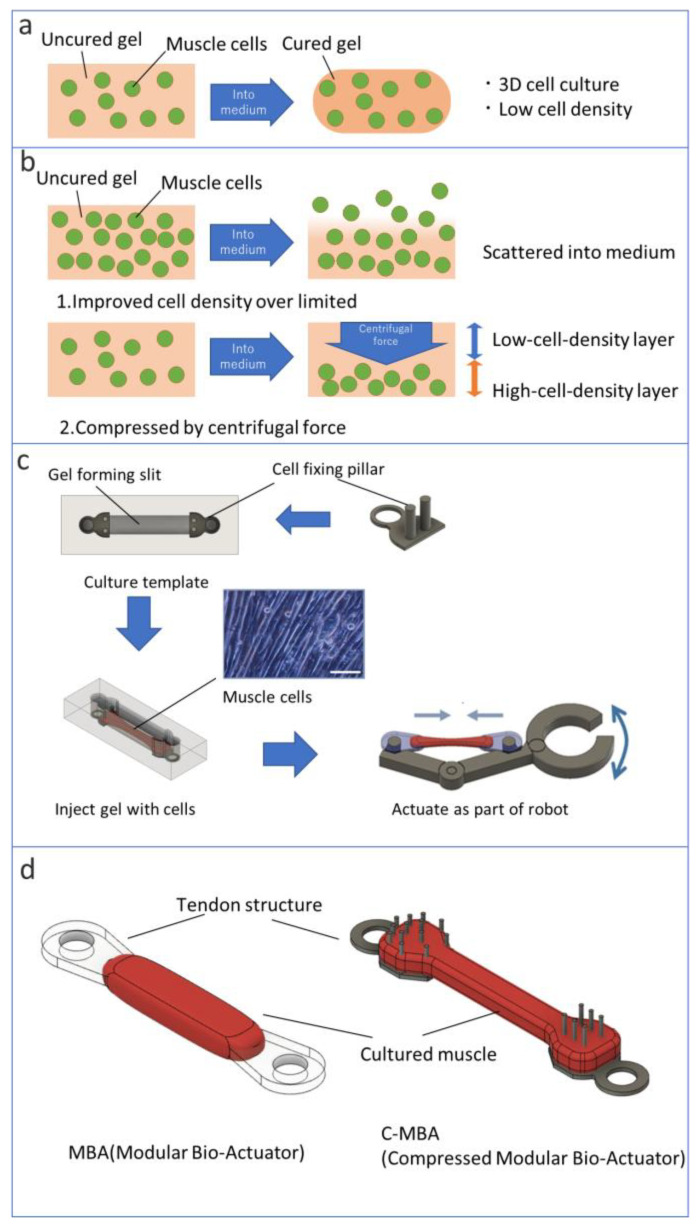
Concept of C-MBA: (**a**) conventional method of fabrication of 3D tissue, (**b**) concepts of centrifugal compressed cell assembling method, (**c**) overview of molding gels to create bioactuators (scale bar: 100 μm), (**d**) overviews of MBA and C-MBA.

Bioactuators have been developed in many studies in the recent decade for actuators of micro-biorobots [2,3,4,5,6,7,8,9,10,11,12,13,14,15]. However, these bioactuators have not shown the same power as animal muscles. The well-known methods for culturing a bioactuator are surface culturing on thin-film shape soft material [2,3,4,5,6,8,9,11,15] or three-dimensional (3D) culture by mixing muscle cells and a scaffold gel [7,16,17,18,19,20,21]. Muscle cells are adherent cells and cannot make a 3D tissue itself. Therefore, scaffold material is needed for muscle cells to form 3D shapes (Figure 1a). Conventionally, the volume of a scaffold gel occupies more than half of the bioactuator. It is one of the reasons the contraction force of bioactuators is less than that of a living muscle. In this paper, we propose the development method for increasing the cell density of a bioactuator using centrifugal force. The research aims at developing a bioactuator with high cell density. By applying the centrifugal force from 0× *g* to 1700× *g* stepwise to the cells’ mixed gel, the cell density of bioactuators was changed, and cell viability under each condition was evaluated. As a result, we selected conditions that could keep high cell viability with high cell density to develop cultured muscles.

Various methods for culturing a cultured muscle in gels under different conditions have been investigated. Among these, M. Costantini et al. investigated the geometrical properties of cell-embedded gels and cell growth rates [22]. They found that the rapid and efficient ability to form myofibers of C2C12 was significantly reduced at high gel stiffness and rigidity, with the best results obtained with hydrogels of the lowest rigidity (~0.33 kPa, GelMA 3). Such reports on 3D culture contrast with previous reports in the literature on 2D culture.

V. Mudera et al. investigated cell density when fabricating a cultured muscle made with a matrix gel [23]. According to these experiments, it can be said that gene expression was helped by high cell density in the initial stage of cell culture by the fabrication of a cultured muscle using a matrix gel. By these results, it can be estimated that forming myofibers with a biological-like structure is improved by high cell density and long culture.

Based on the above, in 3D culture using a matrix gel, reducing the proportion of a matrix gel as much as possible leads to an increase in the contractility of the cultured muscle relative to its unit cross-sectional area. The reduction of the matrix gel leads to a decrease in the strength of the 3D tissue. Creating a 3D tissue without or with reduced use of a gel is challenging.

Yamamoto et al. produced a gel-reduced tissue by compressing magnetically labeled cells with a magnet. This technique is called the magnetic force-based tissue engineering (Mag-TE) technique [24]. Another way to create a tissue without a gel is by collecting spheroids with a metal mesh. Hibino et al. used the method to create a beating myocardial tissue [25]. However, the Mag-TE technique has a complicated process for magnetic labeling, and the method using a metal mesh cannot easily change the shape of the tissue because it uses a metal mesh.

In the method proposed here, the mixture of cells and a matrix gel is centrifugally compressed in the culture template to separate it into a high- and low-cell-density layer. The high-cell-density layer is formed at the bottom of the template, and a low-cell-density layer is formed at the top. The high-cell-density layer becomes a tissue through culture. The low-cell-density layer acts as a lid for the slit in the culture template and is assumed to prevent the collapse of the cultured tissue in the early stages of culture (Figure 1b). We newly propose C-MBA (compressed modular bioactuator), which is an improvement of the already-proposed MBA (modular bioactuator) (Figure 1c,d) [26].

## 2. Materials and Methods

### 2.1. Materials of C-MBA

**Figure 2:**
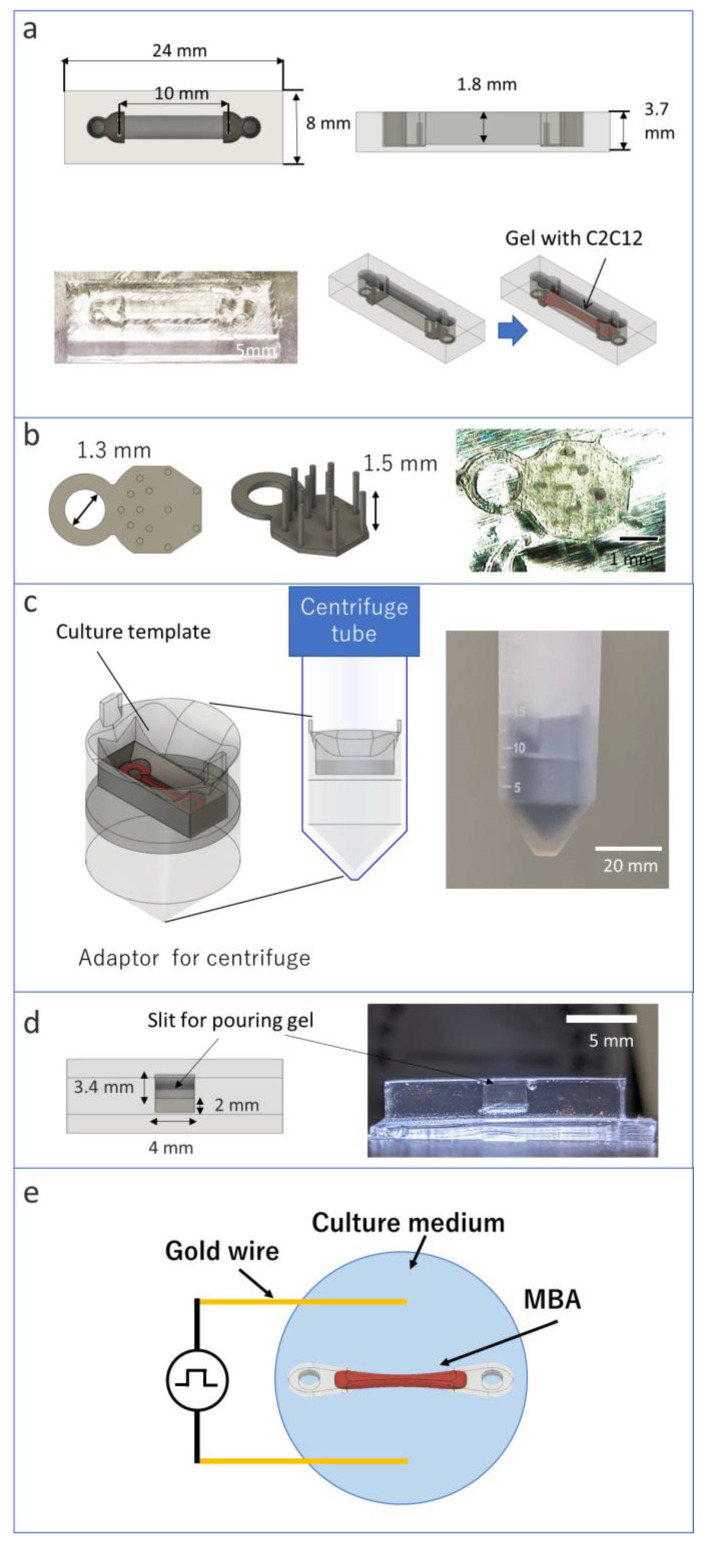
Fabrication of C-MBA’s assembly method: (**a**) culture template, (**b**) tendon structure with some pillars, (**c**) overview of adaptor for centrifuge, (**d**) centrifugal template, (**e**) experimental setup for applying a voltage to the MBA using a gold wire.

This study used C2C12 mouse myoblast cells to fabricate MBAs and C-MBAs. Two types of mediums were prepared for culture cells. A growth medium was used for C2C12 growth in bioactuators, and a multinucleation medium was used for C2C12 cellular differentiation. Both mediums were prepared based on Dulbecco’s Modified Eagle’s Medium (DMEM). Amounts of 100 units/mL of penicillin G, 100 μg/mL of streptomycin sulfate, and 10% fetal bovine serum (FBS) were mixed with DMEM for growth medium. An amount of 2% horse serum (HS) was used as the multinucleation medium instead of 10% FES. A Matrigel (Corning) was used as a scaffold for the 3D culture of cells. It is a basement membrane matrix made from a mouse-derived ECM. The culture template was made of polydimethylsiloxane (PDMS, DuPont Toray Specialty Materials) because PDMS has high biocompatibility and easy processing and molding. Acrylonitrile resin was used for the 3D printing of the molds of culture templates (Figure 2a).

### 2.2. Preparation of Tendon Structures and Culture Template

A sufficient drag force is required for skeletal muscle growth for bioactuators. The cultured skeletal muscle obtains elasticity and tension by cell culture with the drag force. Bioactuators excessively contract by tension without the drag force. Excessively contracted bioactuators cannot have function as an actuator. For these reasons, the muscle should be tightly fixed to apply adequate drag force. Generally, pillar structures have been employed to obtain the drag force during bioactuators’ culture [6].

For the above reasons, the bioactuator must be fixed to the template during the cell culture step. However, both ends of the bioactuator must be connected to a microload cell for force measurement. Therefore, we apply the tendon structure, which was developed as a part of the MBA in the previous study [26]. The tendon structure’s cell anchor part was designed as a comblike structure and can be engaged with a cell tissue. However, applying this cell anchor to the centrifugal compressed cell assembling method is challenging because the cell anchor part needs to be located in the center of the cell tissue. Still, height position control becomes difficult through centrifugal compression. Therefore, many pillars are applied to the tendon structure. A pillar structure has a weak fixation force, but it can fix a thin cell tissue because pillars can fix a cell tissue by piercing the form underside of the cell tissue.

We designed a new tendon structure with a mounting hole and cell anchor (Figure 2b). The mounting hole can be used to fix the culture template during cell culture and replace some sensors. The mounting hole is designed to have a 1.3 mm diameter. The pillars are designed to distribute stress.

The prototype molds of tendon structures and culture templates were designed with 3D CAD. The templates were replicated by PDMS using the mold. PDMS was prepared by mixing the base material and curing agent at a ratio of 10:1. The uncured PDMS was degassed using a vacuum defoamer and desiccator. The degassed PDMS was poured into the molds and placed on a hot plate at 70 °C for about 5 h. The culture template was immersed in 70% ethanol for 1 h, then irradiated with ultraviolet (UV) light for 1 h to sterilize it. The 2-methacryloyloxyethyl phosphorylcholine (MPC) polymer Lipidure-CM 5206 (NOF America Corporation) was coated on the surface of the culture template, and plasma etching treatment was performed to prevent cell adhesion.

The tendon structure was printed by the stereolithography apparatus (SLA) 3D printer Form3 (Form labs). Urethane dimethacrylate (UDMA) was used as the material for the tendon structure. The printed tendon structures were washed with isopropanol and secondarily cured with UV. Furthermore, the tendon structures were immersed in 70% ethanol for 1 h, then irradiated with ultraviolet (UV) light for 1 h for sterilization.

### 2.3. Preparation of Myoblast Cells

The preparation procedure for C2C12 cells was the same as our previous study [26]. C2C12 cells were cultured in a 5% CO_2_ incubator with the growth medium at 37 °C. The subculture was conducted when 80% of the dish’s bottom surface was covered by cells. The dish’s bottom surface was washed with 10% phosphate buffer solution (PBS) after removing the culture medium. An amount of 0.25% trypsin ethylenediamine tetraacetic acid (EDTA) and a cell scraper were employed to detach cells from the dish surface. The centrifugation was performed to collect detached C2C12 cells, and the gathered cells were seeded on a new culture dish.

### 2.4. Attaching Template for Centrifuge

An adaptor was used for attaching the template to the centrifuge. The adaptor was designed with a slit for attaching the template (Figure 2c). The bottom of the template must be vertical to the direction of the centrifugal force to apply the centrifugal force evenly to cells injected into the template. The Kubota centrifuge used in the experiment was equipped with a pendulum mechanism so that the centrifugal force was directed directly below the centrifuge tube. Therefore, the template should be fixed to keep a horizontal position inside the centrifuge tube. The adaptor was designed with the 3D CAD software, and the printing of ABS resin was performed with an FDM 3D printer.

### 2.5. Measuring the Effect of Centrifugal Force on Cells

Before preparing C-MBAs, the effect of the centrifugal force on cells was measured. The centrifugal template was used to measure the effect of the centrifugal force. The centrifugal template was designed to have a 4 mm × 3.4 mm × 2 mm slit for gel injecting (Figure 2d). The outside was designed in common with the culture template. The same adapter was used for compression as well. Volume and cell density can be measured by measuring the height of the compressed cells from the sides of the template. Furthermore, the effect of the centrifugal force on the cells was evaluated by measuring cell viability after the experiment.

Cells were stained with calcein-AM (viable cell stain, Fujifilm Wako Pure Chemicals) and propidium iodide (PI) (dead cell stain, Fujifilm Wako Pure Chemicals) to check the cell viability. The number of viable and dead cells was measured on a cell counterplate to evaluate the viability of the cells after applying centrifugal compression force.

### 2.6. Fabrication of MBA and C-MBA

The fabrication procedure for MBA was the same as described in our previous study [26]. When more than 90% of the dish bottom was covered by C2C12 cells, the cells were used for fabricating MBA. After removing the culture medium, the dish bottom was washed with 10% PBS. An amount of 0.25% trypsin EDTA and a cell scraper detached cells from the dish surface. C2C12 cells were collected in a centrifuge tube by adding the growth medium and pipetting well. The centrifuge tube was put in water at 2 °C for 5 min. The Matrigel and the C2C12 cells were mixed at a 1.5 × 10^8^ cells/mL cell concentration. An amount of 30 μL of the Matrigel with the C2C12 cells was injected into the groove of the culture template. The tendon structure was placed in the culture template, and another 30 μL of the Matrigel with cells was added to the culture template. The Matrigel with cells was cultured in a 5% CO_2_ incubator at 37 °C for 10 min. The cells were cultured for 3 days in the growth medium. The growth medium was exchanged with the multinucleation medium to promote the differentiation of C2C12 cells into myotubes. The multinucleation medium was exchanged every 2 days.

The preparation method for C-MBA is almost the same as that of MBA [26]. When the gel is injected into the template, the template is set into the centrifuge using the adapter. After centrifugal force compression, the template is placed in an incubator to promote gel polymerization. For subsequent culture steps, follow the same steps as that for MBA.

### 2.7. Stimulation for Bioactuator

Electric pulse stimulation was applied to the bioactuator after MBA and C-MBA were cultured over 10 days. A function generator prepared electric pulse stimulation. The parameters of the pulse were as follows: 10 V square wave with 2% duty ratio at 1 Hz frequency. The parameters of electrical pulse stimulation and the direction of the electric field were selected based on previous studies [26,27]. The electric pulse stimulation was applied by conductive wires placed directly into the medium. ϕ 0.5 mm gold wires were used as electrodes (Figure 2e). Gold is suitable for the electrodes because of chemical stability and nonelectrolyzed characteristics.

## 3. Results

### 3.1. Effect of Centrifuge

**Figure 3:**
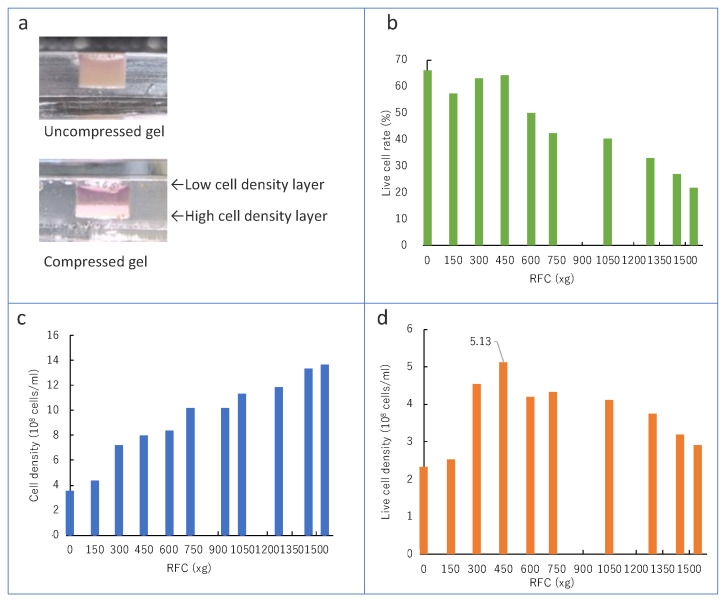
Effect of the centrifugal force on the cell: (**a**) high- and low-cell-density layers separated by the centrifugal force, (**b**) live cell rate changed by the centrifugal force, (**c**) cell density changed by the centrifugal force, (**d**) live cell density inside high cell density changed by the centrifugal force.

The cells in the gel injected into the template were compressed by the centrifugal force and divided into high- and low-cell-density layers, as per the concept (shown in Figure 3a).

Compressibility and cell viability were investigated and evaluated to examine the effect of the centrifugal force on cells. The cell compressibility was evaluated by measuring the cell density of the high-cell-density layer formed by centrifugation. The volume of the high-cell-density layer was calculated by multiplying the bottom area of the template slit by the thickness measured from the side image. The number of cells used in the experiment was measured beforehand using a cell counterplate. The number of cells by unit volume was estimated based on these results and evaluated as cell density. This is because the high-cell-density layer could not be separated from the low-cell-density layer. The high-cell-density layer should be separated from the low-cell-density layer to quantify the cell density after the centrifuge. However, the cells did not form tissues just after the centrifuge. Therefore, the number of cells was measured before centrifuging in this study.

The experimental results of cell density are shown in the graph (Figure 3b). The cell density increased as the centrifugal force increased. It did not reach the upper limit of compression, even at the upper centrifugal force limit of the equipment in this experiment. The maximum value in the experiment was 14.2 × 10^8^ cells/mL.

The change in cell viability as a function of centrifugal force is shown in the graph (Figure 3c). Cell viability also showed a tendency to decrease with an increasing centrifugal force. In this experiment, the lower limit of cell viability was also not reached when the centrifugal force was applied at the upper limit of the equipment. The lowest cell viability in this experiment was 16.1%.

The above results determined the optimal centrifugal force for subsequent compressed cultured muscle production. From the above two graphs, the density of viable cells in the high-cell-density layer at each centrifugal force inferred from the results obtained is shown in the graphs (Figure 3d). As a result, we can say that the viable cell density at 450xg is the highest. Since the peaks in the graph are within the range of the centrifugal forces that were tried, it can be inferred that practical experiments can be performed within the range of the centrifugal forces provided by the equipment used in this experiment. Therefore, from this experiment onward, a parameter of 450xg was used to compress C-MBA.

### 3.2. Fabrication of C-MBA

**Figure 4:**
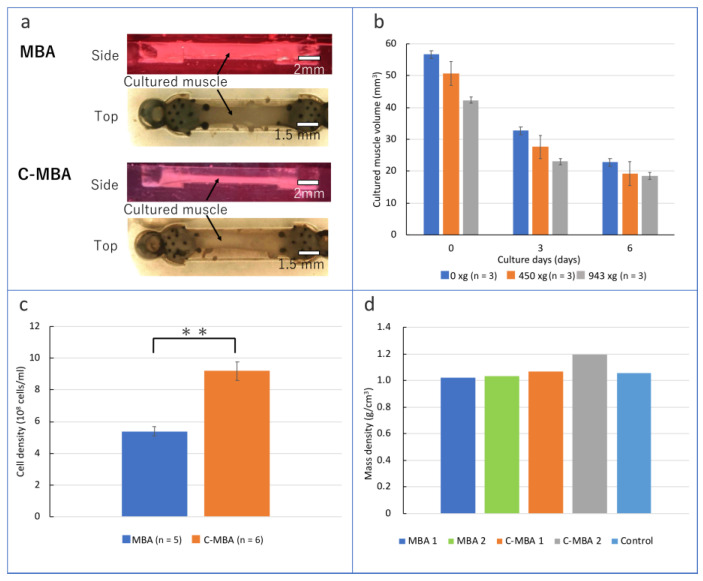
Effect on cultured muscle changed by centrifugal force: (**a**) side and top views of the fabricated MBA and C-MBA, (**b**) changes in volume of cultured muscle during culture, (**c**) cell density of cultured muscle changed by centrifugal force. Asterisks indicate comparable groups and statistical significance (** *p* < 0.01); (**d**) mass density of cultured muscle changed by centrifugal force.

Images of C-MBA fabricated in this study are shown in Figure 4a. The lateral view confirmed that the pillar-type tendon structure held the cultured muscle as designed. A cultured muscle can be cultured for up to 30 days or longer, and the retention force of the tendon structure with pillars is greater than the natural contractility of the cultured muscle.

For the cultured muscle of C-MBA, it was confirmed whether the superiority of cell density at the time of creation was maintained with continued culture. Generally, the cultured muscle shrinks and decreases in volume during the culture period. It is crucial for the C-MBA concept that the cell density advantage at the time of muscle creation is maintained during culture.

Figure 4b shows the cultured muscle’s volume change for the culture period. The cell density advantage during muscle preparation was maintained even after 6 days in culture. However, for samples with a centrifugal force higher than 450× *g*, the cell density was comparable to that of samples with a lower centrifugal force after about 1 week of incubation. In preliminary experiments, the cell viability of individuals centrifuged at 943× *g* was around 40%. The result suggests that the growth of a cultured muscle is inhibited by the low viability of cells inside the cultured muscle.

Next, cell density to volume was measured for C-MBA and MBA that had been in culture for more than 2 weeks. The volumes of C-MBA and MBA used in the experiments were calculated by obtaining top and side photographs. After removing the tendon structures from C-MBA and MBA, the tissue was dissolved in 0.25% TE solution, and the cells were harvested. After adding a culture medium to the harvested cells, the cell volume was measured using a cell counter plate. The C-MBA and MBA cell densities estimated from these measurements are shown in the graph (Figure 4c). The average cell density of C-MBA was 9.2 × 10^8^ cells/mL, while that of MBA was 5.39 × 10^8^ cells/mL, indicating that C-MBA averaged 1.71 times higher cell density than MBA.

### 3.3. Comparison of the Mass Density of C-MBA and MBA

Since the mass density of muscle cells is higher than that of culture media or matrix gels, C-MBAs produced at high cell densities can be expected to show higher values than existing methods. In addition, studies of living muscles often use mass density rather than cell density as the criterion for evaluation. A living muscle contains many cells other than muscle fibers, such as muscle satellite cells, blood components such as red blood cells, and smooth muscle that makes up blood vessels. For this reason, obtaining pure cell density in living muscles is difficult. For these reasons, we measured the mass density of a cultured muscle to compare C-MBA and MBA with a living muscle. The mass was measured by measuring the mass of the entire bioactuator before measuring the cell density and then measuring the mass of the tendon structure after the experiment to obtain the difference.

A comparison of the mass density obtained from two samples for C-MBA and MBA, respectively, is shown in the graph (Figure 4d). In this experiment, C-MBA was made under a 450× *g* condition. The mass density of C-MBA was higher than that of MBA by a maximum of 16.7%. Hence, the difference was not as significant as that of cell density. The tissues prepared in the experiment were compared with the average values of rabbit muscle [28]. The maximum and minimum values were 12.8% and 1.33% higher than living muscles. These values indicate that centrifugal force compression has a strong effect on cell density but a low effect on mass density. The compression did not affect mass density because more than 70% of cells are formed from water. The result that only the cell density is strongly enhanced can be considered as the centrifugal force not being applied to the cells to the extent that the cell walls are destroyed. Therefore, these results can be interpreted as saying that the centrifugal force compresses the cells by pushing out other substances, such as gels and a culture medium.

### 3.4. Contract Force per Unit Cross-Sectional Area

**Figure 5:**
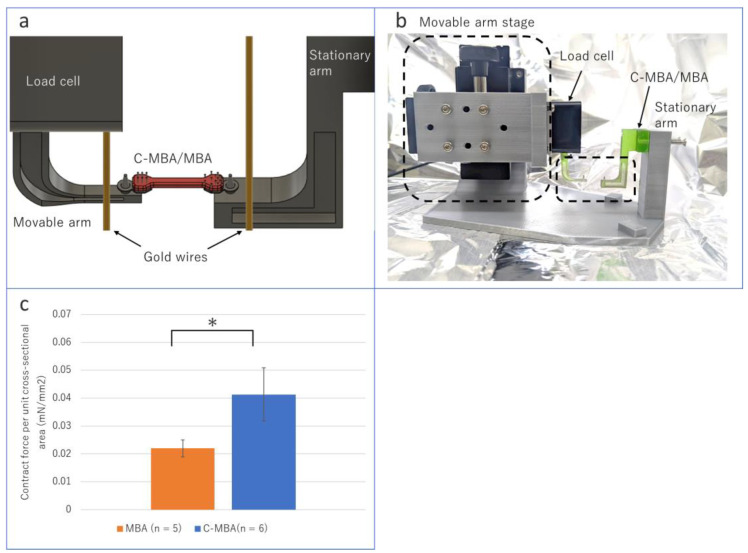
MBA contraction force measurement system: (**a**) measurement system overview, (**b**) actual fabricated measurement system, (**c**) increased contraction force per unit cross-sectional area with C-MBA. Asterisks indicate comparable groups and statistical significance (* *p* < 0.05).

The contractile force was induced by electrical stimulation to evaluate the performance of the cultured muscle. C-MBA and MBA were placed on the system shown in Figure 5a,b via a tendon structure connection. A Kyowa LVS-5GA micro load cell was used to measure the contractile force of the cultured muscle. A gold wire was used to apply electrical stimulation. Gold wires were placed at both ends of the cultured muscle to maximize the potential difference in the cultured muscle. After the experiment, a cross-sectional area was obtained by preparing frozen sections from C-MBA and MBA.

The results of the experiments on C-MBA and MBA after more than 2 weeks of incubation are shown in the graph (Figure 5c). The average contraction force per unit cross-sectional area for C-MBA was 0.041 mN/mm^2^. This value is 1.88 times higher than the average shrinkage force per unit cross-sectional area of 0.022 mN/mm^2^ for MBA. These results show that the centrifugal force reduced the volume of the cultured muscle, reducing its unit cross-sectional area by about half.

## 4. Conclusions

Based on these results, the method proposed in this paper improved the cultured muscle contractility per unit cross-sectional area by centrifugal force compression. The improved contractility per unit cross-sectional area can be rephrased as improved performance when the cultured muscle of the same size is produced. These results indicate that cells compressed by the centrifugal force survive and grow within the cultured muscle. By these results, we estimated that C-MBA could improve the power-to-weight ratio for microrobots. The mass of the actuator is a crucial factor because the microrobot weight is very small. Since a cultured muscle has essentially the same characteristics as a muscle in vivo, its contraction force is proportional to its cross-sectional area. Therefore, if the contraction is not enough for the robot, the cultured muscle must be larger. However, many biorobots are integrated with muscles, so increasing muscle strength needs redesigning. Therefore, it can be said that this study’s fabrication method for the bioactuator can contribute to improving the problem of bio-microrobots: muscle strength relative to weight and size.

Because of the small overall length of the template used in this study, the difference in centrifugal force applied to the center and both ends of the template is expected to be negligible. However, it can be said that an arc-shaped template is necessary for larger tissue fabrication because the centrifugal force on the tissue needs to be equalized.

Compression of a cellular tissue has been applied to compress cardiac muscle sheets by centrifugal force [29,30,31] and atmospheric pressurization of a smooth muscle tissue for blood vessels [32]. Still, its application to bioactuators is less common. The cultured muscle used in bioactuators has been intended for application to robots, not for implantation into living organisms. In most cases, cultured muscles are seeded to cover the joints of a robot and cultured to keep these structures. Since bioactuator systems that include moving parts have complex shapes, applying compression by centrifugal force is challenging. In this study, we solved this problem by applying the previously proposed MBA [26], which can be designed as a separate system for the incubation and operation periods by modularizing the bioactuator. C-MBA allows an optimal template for fabrication and cultivation while creating a completely different system for the working experiments. This culture method has shown biorobotics potential to treat and design a biological tissue as if it were a mechanical component. Such an element will be essential for future biorobot fabrication with even greater degrees of freedom.

Since the method proposed in this study is performed at the very early stage of culture, it is likely to be used in conjunction with existing methods of cultured muscle reinforcement, such as electrical stimulation during culture [33], anisotropy enhancement of muscle fibers by fence geometry [16], and addition of chemicals during culture [34]. However, one of the major remaining issues in such studies is the connection method to the skeleton.

In this study, fixation by pillars applying tendon structures was employed, but the maximum number of pillars for fixation was 13. Therefore, to fabricate cultured muscles with higher output, it is necessary to increase the surface area of the cultured muscle junction, as is the case with many pillars. However, such structures increase the dead weight of the biorobot. In addition, the tendon structure used in MBA is a hard plastic material, different from biological tendons that exhibit moderate elasticity. An example already proposed in these studies is a method of joining a cultured muscle with a spongy material [18], but this also does not have the same elasticity. Another method is to use fibrous material obtained from a venous tissue [17], but the in vivo material is not productive and unsuitable for biorobotic applications. Therefore, in this study, we plan to explore a method of dispersing stress by fabricating a tendon structure similar to a living body rather than a tendon structure made of rigid material.

In this paper, we proposed a method of compressing cell density using centrifugal force and a bioactuator C-MBA fabricated by the method. C-MBA showed 1.71 times higher cell density and 1.88 times higher contractility per unit cross-sectional area than the conventional method. The results of this research can contribute to solving power-to-weight ratio problems in the fields of micro- and biorobotics.

## Data Availability

Not applicable.
